# Evidence supporting oral sensitivity to complex carbohydrates independent of sweet taste sensitivity in humans

**DOI:** 10.1371/journal.pone.0188784

**Published:** 2017-12-27

**Authors:** Julia Y. Q. Low, Kathleen E. Lacy, Robert L. McBride, Russell S. J. Keast

**Affiliations:** 1 Deakin University, Centre for Advanced Sensory Science, School of Exercise and Nutrition Sciences, Burwood, Victoria, Australia; 2 Deakin University, Institute for Physical Activity and Nutrition (IPAN), School of Exercise and Nutrition Sciences, Geelong, Victoria, Australia; Barnard College, UNITED STATES

## Abstract

Compared to simple sugars, complex carbohydrates have been assumed invisible to taste. However, two recent studies proposed that there may be a perceivable taste quality elicited by complex carbohydrates independent of sweet taste. There is precedent with behavioural studies demonstrating that rats are very attracted to complex carbohydrates, and that complex carbohydrates are preferred to simple sugars at low concentrations. This suggests that rats may have independent taste sensors for simple sugars and complex carbohydrates. The aim of this paper is to investigate oral sensitivities of two different classes of complex carbohydrates (a soluble digestible and a soluble non-digestible complex carbohydrate), and to compare these to other caloric and non-nutritive sweeteners in addition to the prototypical tastes using two commonly used psychophysical measures. There were strong correlations between the detection thresholds and mean intensity ratings for complex carbohydrates (maltodextrin, oligofructose) (*r* = 0.94, *P* < 0.001). There were no significant correlations between the detection thresholds of the complex carbohydrates (maltodextrin, oligofructose) and the sweeteners (glucose, fructose, sucralose, Rebaudioside A, erythritol) (all *P* > 0.05). However, moderate correlations were observed between perceived intensities of complex carbohydrates and sweeteners (*r* = 0.48–0.61, *P* < 0.05). These data provide evidence that complex carbohydrates can be sensed in the oral cavity over a range of concentrations independent of sweet taste sensitivity at low concentrations, but with partial overlap with sweet taste intensity at higher concentrations.

## Introduction

Complex carbohydrates and simple sugars are two essential sources of energy in our diet. Except for some fruits, complex carbohydrates are more abundant than simple sugars in plants, but it is sugars with their hedonically pleasing sweet taste that are the sought-after carbohydrate [1]. In line with this, there is also growing evidence demonstrating that rodents (e.g., rats, mice, gerbils, hamsters) and even some non-human primates are attracted to the taste of complex carbohydrates derived from maltodextrin (also known as glucose polymer) [2, 3]. This evidence has been summarised in Lapis et al. [4, 5] indicating independent taste peripheral physiology for complex carbohydrates and simple sugars [3, 6], but the taste receptor remains unknown [7]. Furthermore, recent behavioural studies from exercise science support the notion that humans can detect complex carbohydrates within the oral cavity (see Lapis et al. [4, 5] and systematic review by e Silva et al. [8]).

Two recent human psychophysical studies propose that humans may perceive complex carbohydrates independent of sweet taste (i.e., glucose and sucrose were significantly correlated with each other, but not with complex carbohydrates) [4, 5]. For example, Lapis et al. [5] found that humans (*n* = 25) were able to discriminate complex carbohydrate solutions (glucose oligomers but not glucose polymers) from water even when the sweet taste receptors (T1R2-T1R3 heterodimer) are inhibited by lactisole treatment. Lactisole is a sweet taste blocker known to bind to a pocket in the transmembrane region of the T1R3 and thus inhibits the sweet taste perception of sugars, proteins, and non-nutritive sweeteners. [5, 9] While the human taste perception of complex carbohydrate (starch) has been investigated by Lapis et al. [4, 5], it needs replication and also extension.

At present, there is also only one known human psychophysical study that has investigated if oral sensitivity to complex carbohydrates is independent of some of the other basic tastes (i.e., sweet and salty taste). Lapis et al. [4] showed no significant correlations between the intensity ratings of glucose (sweet taste), sucrose (sweet taste), and sodium chloride (salty taste) with the intensity ratings of complex carbohydrates. However, it is still uncertain if this measure is independent of the intensity ratings of the remaining common prototypical tastes stimuli such as monosodium glutamate (umami taste), caffeine (bitter taste), and citric acid (sour taste). As each measure of taste function (detection threshold, recognition threshold, and suprathreshold intensity perception) represents a different dimension of the sense of taste, there is currently no single method to measure taste function in totality [10, 11]. Even though the perceptual relationship between a range of caloric and non-nutritive sweeteners have been reported, there is currently no single study that has investigated the relationships between complex carbohydrates and multiple sweeteners (caloric and non-nutritive) using a range of psychophysical measures within a single group of individuals. It is also important to test other stimuli within a single class to access similarities in terms of perception between soluble digestible and soluble non-digestible complex carbohydrates.

The aim of this paper was to investigate if humans can perceive two different classes of complex carbohydrates (a soluble digestible and a soluble non-digestible carbohydrate), and to associate the oral sensitivities of these complex carbohydrates to other caloric and non-nutritive sweeteners using two commonly used psychophysical measures. Although the terminology “polysaccharide taste” has been recommended by Sclafani [1] to denote starch-derived saccharides containing three or more glucose units, it can be confusing as the word “polysaccharide” is generally used to describe complex carbohydrates, comprising more than ten monosaccharide units organised in chains. The word “oligosaccharide taste” (two to nine monosaccharide units) would be the more appropriate terminology, nonetheless, and it is not user friendly. However, at the present time, it cannot be confirmed if perception of oligosaccharides is independent of textural differences. Therefore, at this stage of knowledge we use “oral sensitivity to complex carbohydrate”, which correctly comprises all types of complex carbohydrates and derivatives including fibres (e.g., oligofructose), while not diminishing the prospect that oral perception of complex carbohydrates could be due to textural differences. Whilst dietary “carbohydrate” is an umbrella term for the monosaccharide and disaccharide sugars as well as starches and fibres, the term “sweet taste” has been collectively used to indicate sweetness. Thus “oral sensitivity to complex carbohydrate” would at the current state of knowledge be as correct as possible without oversimplifying tasting complex carbohydrates, but not easily confused with other sensations such as sweetness.

## Materials and methods

### Study design

This study comprised a total of 28 laboratory-based sessions in which data on two measures of taste perception routinely used in chemosensory research was collected: (1) detection threshold (DT) and (2) suprathreshold intensity rating (ST). These measures were determined for all participants for each of two complex carbohydrates (soluble digestible and non-digestible complex carbohydrate), six sweeteners (caloric and non-nutritive sweeteners) and prototypical stimuli for sour, salty, umami, and bitter. The 28 laboratory-based sessions consisted of 14 testing sessions to measure oral complex carbohydrate sensitivity (seven repeated testing sessions for each complex carbohydrate); 12 sessions to measure sweet taste function (each sweetener measurements collected over two repeated testing sessions); one session to measure the prototypical stimuli (each measurement collected in duplicates in a single testing session); and one session for general Labeled Magnitude Scale (gLMS) training and standardisation. All repeated testing sessions were separated by at least an hour apart. For DT measures, if there were more than three concentration step differences between the repeated measures, participants attended another session to complete the assessment. Participants in the present study were part of a larger study focusing on the psychophysics of sweet taste measures for the six sweeteners [12]. The present study shared the same dataset (sweet taste function only) with Low et al. [12]. DT and ST tasks were conducted in computerised, partitioned sensory booths in the Centre for Advanced Sensory Science using Compusense Cloud Software as part of the Compusense Academic Consortium (Compusense Inc., Ontario, Canada). Filtered deionised water was used as an oral rinsing agent. Participants were instructed to rinse their mouths with filtered deionised water for five seconds before beginning each task and between each sample set. To eliminate any potential visual and olfactory input, all testing sessions were conducted under red lighting, and participants were asked to wear nose clips during testing. All solutions were served at room temperature, with a three-digit code allocated to each sample.

### Participants

Participants [(*n* = 34): 16 males, age 26.2 ± 0.4 years (range, 24–30 years), BMI 25.2 ± 0.9 kg/m^2^ (range, 18.9–30.0 kg/m^2^); 18 females, age 29.4 ± 2.1 years (range, 24–55 years), BMI 24.3 ± 0.8 kg/m^2^ (range, 20.0–29.6 kg/m^2^)] were recruited via email and flyer distribution from locations adjacent to the Melbourne Burwood campus of Deakin University, Australia. Potential participants were excluded if they were: (1) smokers; (2) pregnant or lactating; (3) taking any prescription medication that may interfere with their ability to taste; or (4) had a history of food allergies that may interfere with the study. Participants were asked to refrain from eating, drinking (except room temperature water), brushing their teeth, and chewing gum for one hour prior to testing. All participants gave written informed consent and were compensated for their participation. This study was approved by the institutional review board regulations of Deakin University (HEAG_H_182_2014) and recruitment started from 02/03/2015-30/09/2015. The experimental protocol was also registered under the Australian New Zealand Clinical Trials Registry (ACTRN12616001356459), www.anzctr.org.au. This study also complies with the Declaration of Helsinki for Medical Research involving Human Subjects.

### Participant training

Prior to using the general Labeled Magnitude Scale (gLMS) to rate taste intensity, participants were trained using the standard protocol outlined by Green et al. [13, 14] except the top of the scale was described as the strongest imaginable sensation of any kind [15]. The 100-point scale comprised the following adjectives: ‘no sensation’ = 0, ‘barely detectable’ = 1.5, ‘weak’ = 6, ‘moderate’ = 17, ‘strong’ = 35, ‘very strong’ = 52, and ‘strongest imaginable’ = 100 [15]. Scales with only adjectives (not numbers) were presented to participants. During the training session, participants were taught to rate the intensity of the perceived sensation relative to a remembered or imagined sensation when using the gLMS scale. Participants were required to rate a list of seven remembered or imagined sensations, such as the warmth of the lukewarm water, the pain from biting of the tongue, and the sweetness of fairy floss (known as cotton candy in the USA, or candy floss in the UK).

### Stimuli

Maltodextrin and oligofructose were used to investigate oral complex carbohydrate sensitivity (DTs and STs for both complex carbohydrates; for details of stimuli see Table 1). Maltodextrin with a dextrose equivalent (DE) of five was used in this study as it contains the lowest possible amount of free sugar (glucose, maltose) yet is soluble in water. DE is a measure of the percentage of reducing sugars relative to glucose on a dry basis [16].

**Table 1:** Complex carbohydrate and sweetener concentrations used for determination of detection thresholds.

Stimulus	Concentration (% *w/v*)											
	1	2	3	4	5	6	7	8	9	10	11	12
**Maltodextrin**	0.04	0.06	0.1	0.2	0.3	0.6	1.1	1.9	3.6	6.3	11.2	20.0
***Amount of Glucose in Maltodextrin (10***^***−3***^***)***	0.3	0.5	0.9	1.6	2.8	5.8	9.0	15.9	28.4	50.5	90.0	160.0
***Amount of Total Sugars in Maltodextrin (10***^***−3***^***)***	1.1	1.6	3.0	5.6	9.8	17.6	31.4	55.7	99.4	176.7	314.7	560.0
**Oligofructose**	0.04	0.06	0.1	0.2	0.3	0.6	1.1	1.9	3.6	6.3	11.2	20.0
***Amount of Fructose in Oligofructose (10***^***−3***^***)***	0.5	0.8	1.5	2.8	4.9	8.8	15.6	27.8	49.7	88.3	157.3	280.0
***Amount of Total Sugars in Oligofructose (10***^***−3***^***)***	1.2	1.8	3.3	6.6	10.5	18.9	33.6	59.7	106.5	189.3	337.2	600.0
**Glucose**	0.02	0.03	0.05	0.09	0.1	0.2	0.4	0.6	1.1	1.8	2.9	4.8
**Fructose**	0.01	0.02	0.03	0.05	0.08	0.1	0.2	0.3	0.5	0.9	1.5	2.5
**Sucrose**	0.01	0.02	0.03	0.06	0.09	0.1	0.2	0.4	0.7	1.2	1.8	3.0
**Sucralose (10**^**−3**^**)**	0.02	0.04	0.06	0.09	0.1	0.2	0.4	0.7	1.1	1.9	3.1	5.1
**Rebaudioside A (10**^**−3**^**)**	0.03	0.05	0.09	0.1	0.2	0.3	0.6	1.0	1.7	2.8	4.6	7.7
**Erythritol**	0.02	0.03	0.05	0.08	0.1	0.2	0.3	0.6	0.9	1.6	2.6	4.4

The concentration series for sucrose was adapted from ISO3972 [17]. The concentration series for maltodextrin, oligofructose, glucose, fructose, sucralose, erythritol, and Rebaudioside A were prepared with successive 0.25 log dilution steps. Reference chemical details: maltodextrin (Star-Dri 5, Tate & Lyle Ingredients Americas, USA); oligofructose (Fibrulose F97, CoSucra-Groupe Warcoing, Belgium); glucose (The Melbourne Food Depot, Melbourne, Australia); fructose (The Melbourne Food Depot, Melbourne, Australia); sucrose (CSR, Yarraville, Australia); sucralose (The Melbourne Food Depot, Melbourne, Australia); Rebaudioside A (AuSweet, Melbourne, Australia); and erythritol (AuSweet, Melbourne, Australia). Calculation of the amount of common and total sugars in maltodextrin and oligofructose concentrations were according to the report of analysis by the Australian Government National Measurement Institute from samples used in this study, where there were a total of 2.8g/100g (2.8% *w/w)* of free sugars for the maltodextrin (Glucose: 0.8% *w/w*) and 3.0g/100g (3.0% *w/w*) of free sugars for the oligofructose (Fructose: 1.4% *w/w*).

Both caloric (glucose, fructose, sucrose, and erythritol) and non-nutritive sweeteners (NNS) (sucralose and Rebaudioside A) were used to investigate sweet taste (for details of stimuli see Table 1). Prototypical stimuli [sodium chloride (Saxa, Premier Foods Inc, Seven Hills, Australia), citric acid (Ward McKenzie Private Limited, Altona, Australia), caffeine (Sigma Aldrich, Steinham, Germany), and monosodium glutamate (MSG; Ajinomoto Cooperation, Tokyo, Japan)] were used to investigate taste function for salty, sour, bitter, and umami. All samples were prepared fresh on the day of testing using filtered deionised water (Cuno Filter Systems FS117S, Meriden, CT, USA) and stored in glass beakers at room temperature (20 ± 1°C).

#### Analysis of common sugars in maltodextrin and oligofructose samples

To determine if the maltodextrin and oligofructose used in this study would be suitable products, four percent *w/v* maltodextrin and oligofructose solutions were prepared for High Performance Liquid Chromatography (HPLC).

The complex carbohydrate extracts were clarified with 25mL acetonitrile and filtered through a 0.45um filter into a 2mL vial. To determine the amount of common sugars in samples, filtered solutions were analysed by HPLC using amino column with an acetonitrile/water mobile phase containing salt and refractive index detection. Quantitation was made using a standard solution containing known amount of fructose, glucose, sucrose, maltose and lactose. Samples were measured in duplicate.

There were a total of 2.8g/100g (2.8% *w/w*) of free sugars for the maltodextrin (Glucose: 0.8% *w/w*) and 3.0g/100g (3.0% *w/w*) of free sugars for the oligofructose (Fructose: 1.4% w/w) used in this study (Table 2). Detailed in Table 1 are the amounts of common sugars and total sugars (% w/v) present in each complex carbohydrate DT concentration.

**Table 2:** Saccharide composition of the oligosaccharides used in the present study.

Proximates	Sample Reference (% *w/w*)	
	Maltodextrin	Oligofructose
**Glucose**	0.8	< 0.2
**Fructose**	< 0.2	1.4
**Sucrose**	< 0.2	1.3
**Maltose**	0.9	< 0.2
**Lactose**	< 0.2	< 0.2
**Maltotriose**	1.1	< 0.2
**Galactose**	< 0.2	< 0.2
Total sugars*	2.8	3.0

*Total Sugars = Glucose, Fructose, Sucrose, Maltose, Lactose, Maltotriose, and Galactose.

These analyses were determined by the Australian Government National Measurement Institute, and were conducted by High Performance Liquid Chromatography (HPLC). 20g of each sample were sent for analyses.

### Detection threshold determination for sweet taste and oral sensitivity to complex carbohydrates

Detailed in Table 1 are the concentration ranges used to assess DT for sweet taste and oral complex carbohydrate sensitivity. The concentration series for sucrose was adapted from ISO3972 [17]; concentrations for the remaining sweeteners and complex carbohydrates were prepared with successive 0.25 log dilution steps [12]. Initial starting concentrations for sweeteners were determined through informal bench-top testing, based on modified findings of matching sweetness intensity ratios published by Keast et al. [18]. Concentrations for complex carbohydrates were derived based on previous published findings of perceptually distinctive oral sensation concentrations (i.e., see Lapis et al. [4] and systematic literature review by e Silva et al. [8]) and without perceivable viscosity. After pilot testing, a concentration range between 0.04–20.0 percent (*w/v*) was used to measure DT levels for complex carbohydrates. As maltodextrin is similar in oral sensation and appearance to oligofructose, similar concentrations were used for both complex carbohydrates [19–22]. DTs for each of the sweeteners and complex carbohydrates were determined using ascending forced choice triangle methodology [23, 24], in which the participants were provided with sets of three 25 mL samples, two of which were controls (filtered deionised water) and one contained sweetener/complex carbohydrate, in ascending order from the lowest to the highest concentration level. Participants were instructed to select the ‘odd’ one out which contained a fixed concentration of particular sweetener/complex carbohydrate. If the participant was incorrect, a second sample set with the next highest concentration of sweetener/complex carbohydrate was presented. However, if correct, a second set was presented with the same concentration as the preceding tray. This continued until the participant could identify the odd sample correctly for three consecutive times. DT was defined as the concentration of sweetener/complex carbohydrate required for a participant to correctly identify the sweetened/complex carbohydrate sample as the odd one out in three consecutive sample sets at one concentration level [23].

### Detection threshold determination for salty, sour, bitter, and umami taste

DT was determined using the procedure outlined in the International Standards Organisation (ISO) Method of Investigating Sensitivity of Taste [17]. Nine concentrations were used for each taste quality with the ninth concentration being presented only when participants were unable to differentiate the solutions from water in the previous eight concentrations [concentration ranges: sodium chloride (salty) 0.01–0.33% *w/v*; caffeine (bitter) 0.006–0.045% *w/v*; citric acid (sour) 0.013–0.10% *w/v*; and MSG (umami) 0.008–0.17% *w/v*] [17]. The eight samples from each taste quality were served in ascending concentration (15 mL per sample, in accordance with the standard ISO method), and each taste quality was presented to participants independently. Participants were unaware of the presentation order but were informed of the possible taste qualities. Participants were instructed to taste each sample for five seconds then expectorate and record whether: there was an absence of taste (water-like); a taste was identified but not recognised; or a taste quality was perceived [17]. DT was defined as the concentration at which the participants selected the ‘taste identified, but unknown taste quality’ response [17].

### Suprathreshold intensity ratings for the sweeteners, complex carbohydrates, and prototypical tastants

Three concentrations (weak, medium, and strong) and a control (blank) solution were prepared to determine perceived ST for each prototypical tastant and sweetener (Table 3). For complex carbohydrates, four concentrations of complex carbohydrate solutions [weak (3.6% *w/v*), medium (6.3% *w/v*), medium-strong (11.2% *w/v*), strong intensity (20.0% *w/v*)] and a control (blank) solution were prepared. These concentrations were derived through informal bench-top testing (ascending taste intensity), but were similar to the concentrations outlined by Webb et al. [12]. The concentrations for each stimulus ranged from “weak” to “strong” on the gLMS. Each stimulus was presented to participants independently (sets), but in a randomised order.

**Table 3:** Concentrations (weak, medium, and strong intensity) of prototypical tastants and sweeteners used for determination of suprathreshold taste intensity.

Taste quality	Stimulus	Concentration (% *w/v*)		
		Weak	Medium	Strong
**Salty**	**Sodium chloride**	0.6	1.2	2.3
**Bitter**	**Caffeine**	0.02	0.04	0.08
**Sour**	**Citric acid**	0.02	0.06	0.13
**Umami**	**Monosodium glutamate (MSG)**	0.05	0.10	0.20
**Sweet**	**Glucose**	5.3	10.6	21.2
**Sweet**	**Fructose**	2.9	5.6	11.2
**Sweet**	**Sucrose**	3.4	6.9	13.7
**Sweet**	**Sucralose (10**^**−3**^**)**	5.7	11.4	22.8
**Sweet**	**Rebaudioside A (10**^**−3**^**)**	8.6	17.2	34.4
**Sweet**	**Erythritol**	5.7	9.8	19.7

### Standardisation of gLMS usage with weight ratings

To standardise gLMS usage within participants, a modified version of the method used by Delwiche et al. [25] was adapted for this study. To control for idiosyncratic scale usage, participants were asked to rate the heaviness of six, visually identical weights (opaque bottles filled with sand and stone and completely wrapped in aluminium foil; weights of 53, 251, 499, 724, 897, and 1127g). Participants were asked to hold out their non-dominant hand palm up, while the experimenter placed the weighted bottle on the palm of the hand. Participants were instructed to rate the heaviness of each weight using the gLMS.

There was a significant correlation between the overall mean prototypical ratings and overall mean heaviness ratings (*r* = 0.39, *P* < 0.05). Assuming that the intensity ratings of prototypical tastants and the heaviness of the bottles were unrelated, the significant correlation indicates that the gLMS ratings were subject to differences in individual scale-use and thus require standardisation across participants [10, 11, 25]. To determine a personal standardisation factor, the grand mean for heaviness across weight levels and participants was divided by each participant’s average intensity for heaviness [11]. Each individual’s prototypical taste intensity and sweetness intensity ratings were multiplied by his or her personal standardisation factor for scale-use bias [11, 25].

### Statistical analysis

Statistical analysis was performed using IBM SPSS statistical software version 23.0 (SPSS, Chicago, IL, USA). Data are presented as means with standard errors of mean (SEM). The DTs and STs were determined as the arithmetic mean of the repeated measures, and Intraclass Correlation Coefficient (ICC) was used as an indicator of reliability. For STs, the geometric mean score of the three/four ratings (weak, medium, medium-strong, and strong) was calculated [10]. Spearman’s rank correlation coefficient was calculated between distinct measures of taste function. In order to simplify the data presentation for correlations between DTs and STs, negative *r*-values were converted to positive and vice versa [11, 12]. The criterion for statistical significance was set at *P* < 0.05.

DTs for each complex carbohydrate, sweetener, and prototypical tastant were treated as grouping variables (tertiles) with participants categorised as more sensitive (1/3), normal sensitive (2/3), and less sensitive (3/3) to explore relationships between oral complex carbohydrate sensitivity, sweet taste function, and prototypical taste function. STs for each complex carbohydrate, sweetener, and prototypical tastant were treated as grouping variables (tertiles) with participants categorised as those who experienced low intensity (1/3), moderate intensity (2/3), and high intensity (3/3) to explore relationships between oral complex carbohydrate sensitivity, sweet taste function, and prototypical taste function. DTs and STs for each complex carbohydrate, sweetener, and prototypical tastant were grouped into tertiles to allow comparison of most and least sensitive groupings or those groups who experienced low and high intensity (i.e., 24 sets of tertiles were determined: one for DT for each complex carbohydrate, sweetener, and prototypical tastant, and one for ST for each complex carbohydrate, sweetener, and prototypical tastant).

## Results

### Test-retest reliability of complex carbohydrates

All measured thresholds and suprathreshold intensities proved reliable. For maltodextrin, the test-retest correlation reached significance for both detection [*r* = 0.91–0.95 (ICC = 0.95), *P* < 0.001] and suprathreshold intensity perception [*r* = 0.50–0.98 (ICC = 0.66–0.85), *P* < 0.001]. Similarly, for oligofructose, the test-retest correlation reached significance for both detection [*r* = 0.88–0.97 (ICC = 0.95), *P* < 0.001] and suprathreshold intensity perception [*r* = 0.47–0.96 (ICC = 0.51–0.94), *P* < 0.001].

### Oral detection thresholds of complex carbohydrates and relationship with taste detection thresholds of sweeteners

**Fig 1:**
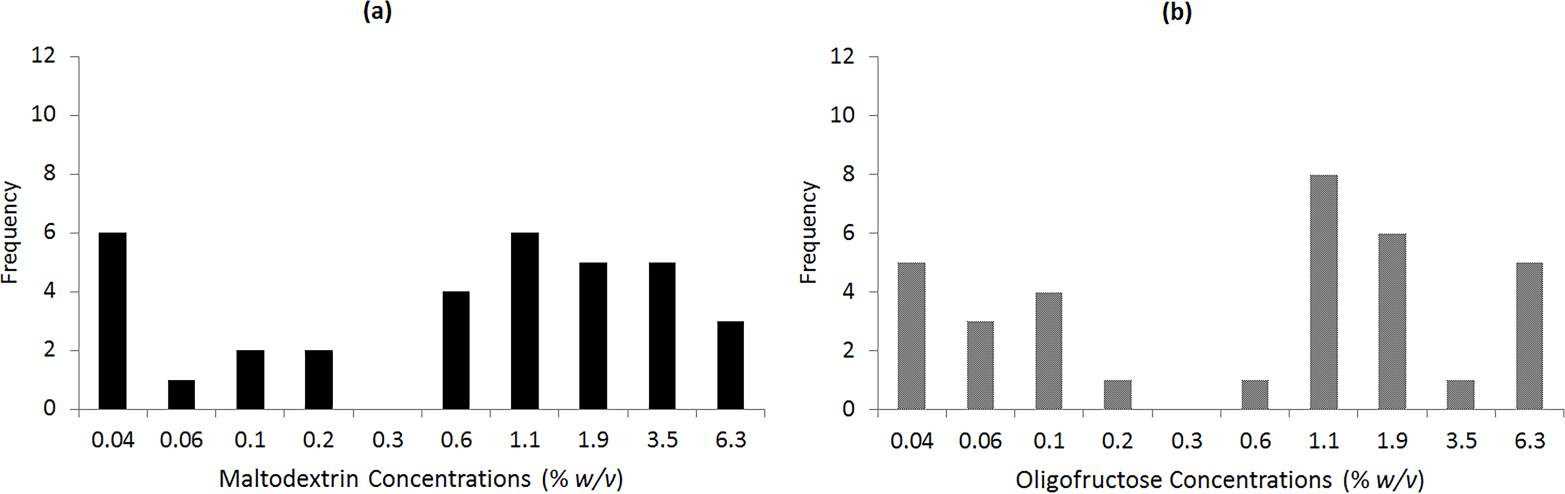
Frequency distributions of detection thresholds. (a) maltodextrin, (b) oligofructose.

There were no significant differences in both oral complex carbohydrate sensitivity and sweet taste function between male and female participants; therefore, the data are presented together (all *P* > 0.05). Mean (± SEM) DT values for the complex carbohydrates are presented in Table 4. There was large individual variation among the participants, for example DT for maltodextrin ranged from 0.04 to 6.31% *w/v* (Fig 1). Mean DT values and frequency distribution of DTs for the six sweeteners has previously been published in Low et al. [12].

**Table 4:** Detection thresholds for complex carbohydrates (% *w/v*), including mean, standard error of mean (SEM), and range.

	Mean ± SEM	Range
**Maltodextrin**	1.7 ± 0.3	0.04–6.3
**Oligofructose**	1.8 ± 0.4	0.04–7.7

**Fig 2:**
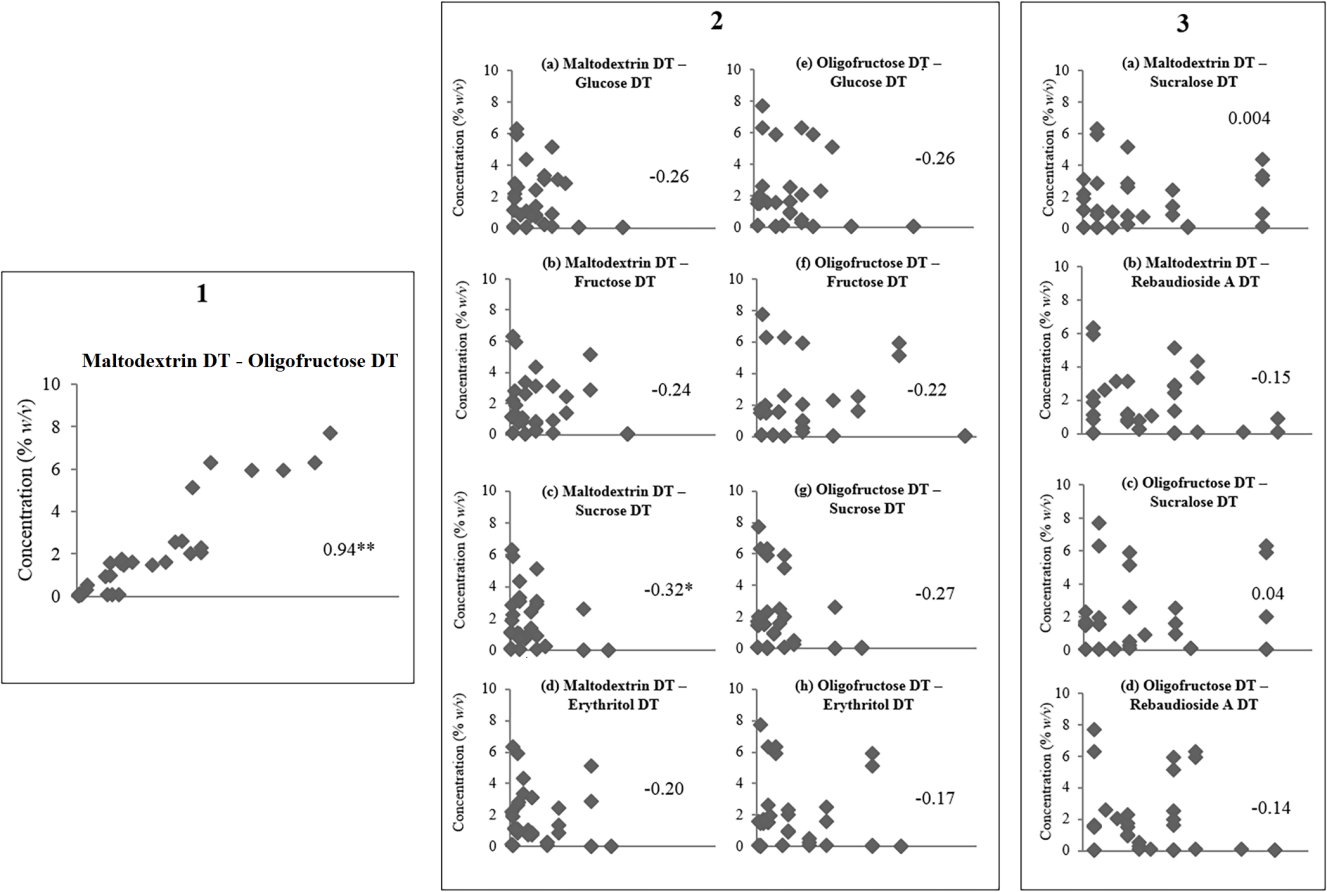
Spearman rank correlations between detection thresholds (DTs) of complex carbohydrates and sweeteners. (1) Spearman rank correlations between detection thresholds of maltodextrin and oligofructose. (2a-d) Correlations between detection thresholds of maltodextrin and caloric sweeteners: (2a) glucose; (2b) fructose; (2c) sucrose; (2d) erythritol. (2e-h) Correlations between detection thresholds of oligofructose and caloric sweeteners: (2e) glucose; (2f) fructose; (2g) sucrose; (2h) erythritol. (3a, 3b) Correlations between detection thresholds of maltodextrin and non-nutritive sweeteners: (3a) sucralose; (3b) Rebaudioside A. (3c, 3d) Correlations between detection thresholds of oligofructose and non-nutritive sweeteners: (3c) sucralose; (3d) Rebaudioside A. *P < 0.05; **P < 0.001.

The DTs of complex carbohydrates (maltodextrin, oligofructose) were strongly correlated with one another (*r* = 0.94, *P* < 0.001; Table 4; Fig 2). Similarly, caloric sweeteners (glucose, fructose, sucrose, and erythritol) were strongly correlated with one another (*r* = 0.84–0.93, *P* < 0.001), as were NNS (sucralose, Rebaudioside A) (*r* = 0.68, *P* < 0.001) [12]. To verify that free sugars in complex carbohydrate solutions were below DT, if a participant is able to detect glucose in water (DT) at the lowest concentration (0.02% *w/v*), potentially that would trigger detection for maltodextrin solution at step 6 (total sugars in maltodextrin: 0.018% *w/v*). However, there were no significant correlations between the DTs of the complex carbohydrates (maltodextrin, oligofructose) and the sweeteners (glucose, fructose, sucralose, Rebaudioside A, erythritol) (all *P* > 0.05) [12]. This suggests that threshold sensitivity to complex carbohydrates (maltodextrin, oligofructose) does not predicate that the person will be sensitive to the sweetness of sweeteners.

### Suprathreshold intensities for the complex carbohydrates and relationship with measures of taste function

**Fig 3:**
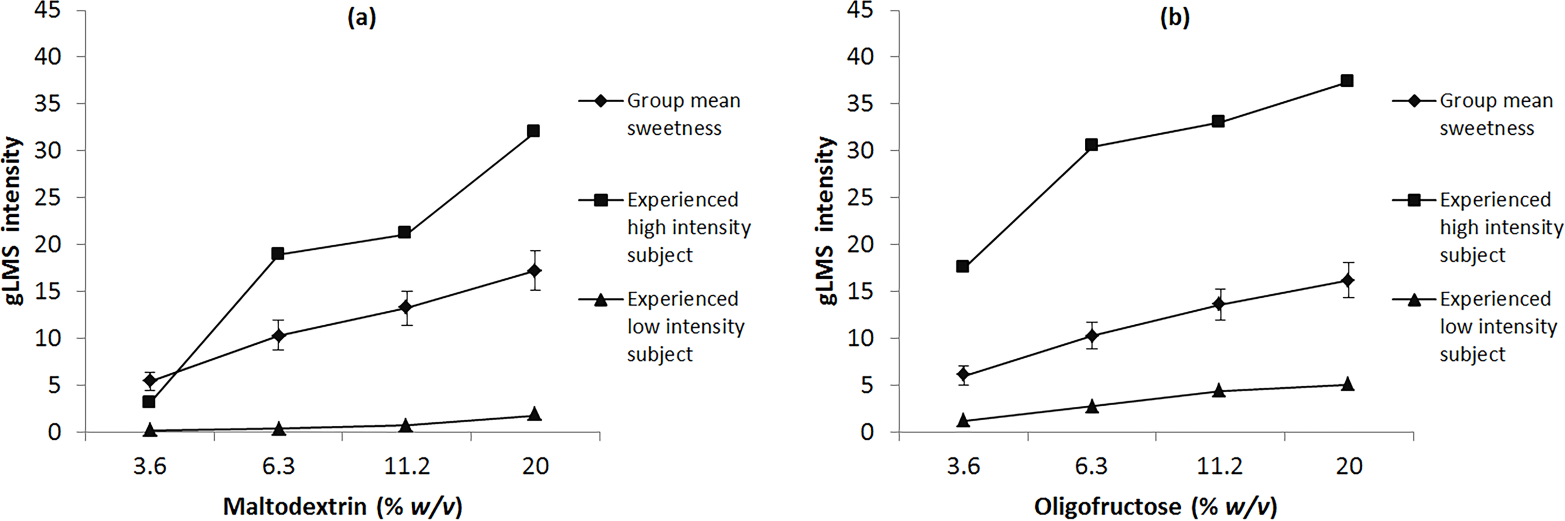
Psychophysical curves of the group mean and examples of a participant who experienced high intensity and a participant who experienced low intensity. (a) Maltodextrin (b) Oligofructose. Included in each graph is the mean psychophysical curve as well as an example of a participant who experienced high intensity (highest curve) and a participant who experienced low intensity (lowest curve) for that complex carbohydrate. The y-axis is a numerical measure of intensity perception from the gLMS. The x-axis is the actual concentration in % *w/v*.

**Fig 4:**
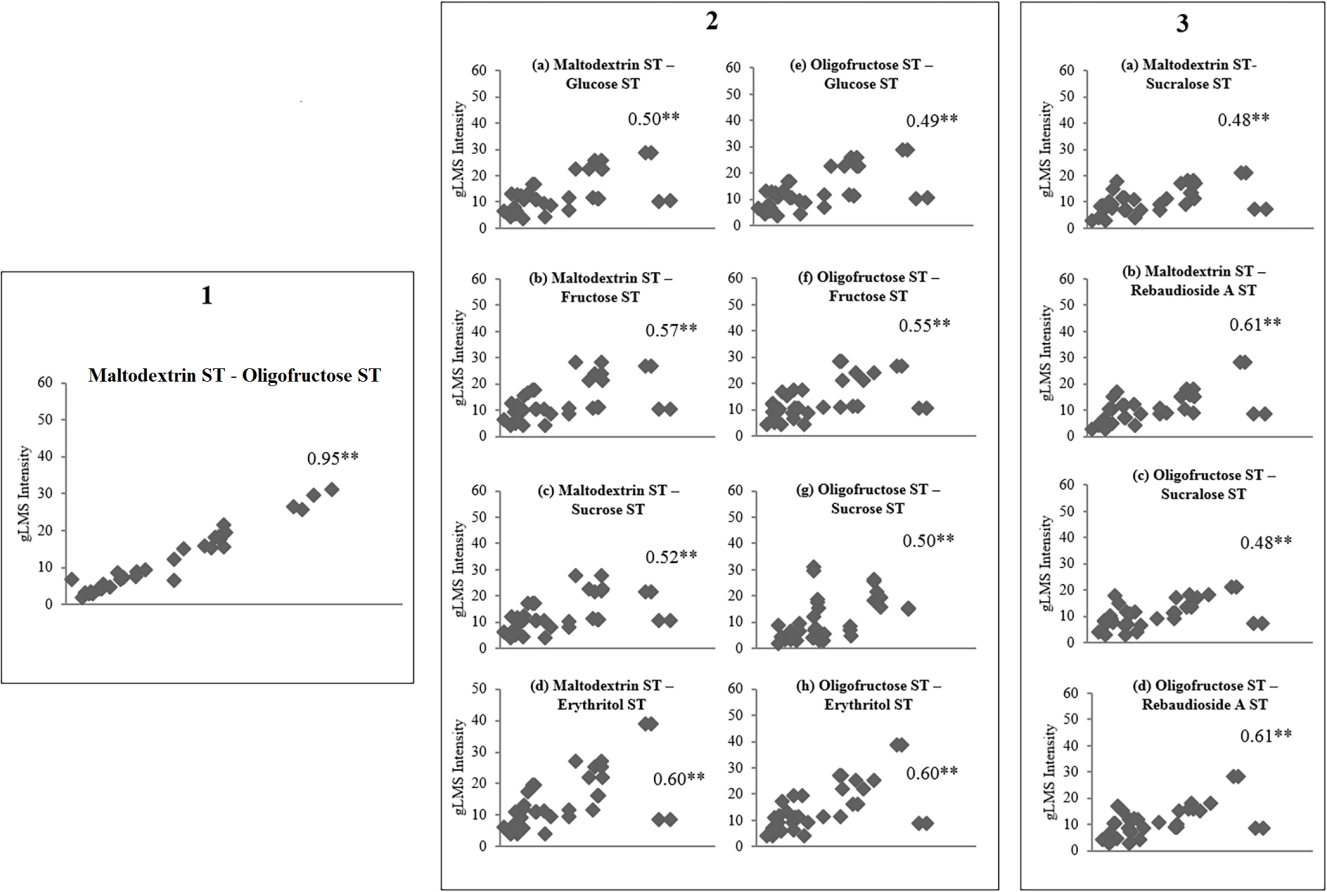
Spearman rank correlations of suprathreshold intensity ratings (STs) between complex carbohydrates and sweeteners. Spearman rank correlations of intensity ratings between maltodextrin and oligofructose. (2a-d) Correlations between intensity ratings of maltodextrin and caloric sweeteners: (2a) glucose; (2b) fructose; (2c) sucrose; (2d) erythritol. (2e-h) Correlations between intensity ratings of oligofructose and caloric sweeteners: (2e) glucose; (2f) fructose; (2g) sucrose; (2h) erythritol. (3a, 3b) Correlations between intensity ratings of maltodextrin and non-nutritive sweeteners: (3a) sucralose; (3b) Rebaudioside A. (3c, 3d) Correlations between intensity ratings of oligofructose and non-nutritive sweeteners: (3c) sucralose; (3d) Rebaudioside A. **P < 0.001.

Fig 3 shows the psychophysical functions for both complex carbohydrates. Psychophysical functions for the six sweeteners have previously been published in Low et al. [12]. As expected there were monotonic increases in perceived intensity as the concentration of the stimuli was increased. Spearman’s rank correlation revealed a significant relationship between the STs at the four concentrations on a complex carbohydrates’ psychophysical function: (maltodextrin *r* = 0.77–0.92, *P* < 0.001); (oligofructose *r* = 0.76–0.95, *P* < 0.001). Analysis of variance showed significant differences between all incremental steps on the psychophysical functions (*P* <0.05). This indicates that when a participant is given increasing concentration of a complex carbohydrate (above the DT); there is an ordinal increase in intensity relative to STs across all participants. For each participant, there were strong correlations between the mean STs of complex carbohydrates (maltodextrin, oligofructose) (*r* = 0.95, *P* < 0.001; Fig 4). There were also moderate correlations between the geometric mean of the STs of complex carbohydrates and sweeteners (*r* = 0.48–0.61, *P* < 0.05). Significant correlations were observed between DTs and STs for maltodextrin and oligofructose (r = 0.39–0.53, *P* < 0.05).

### Taste function of prototypical tastants and relationships between oral complex carbohydrate sensitivity, sweet taste function, and prototypical taste function

DTs and STs of the four-prototypical tastes are presented in Tables 5 and 6. No robust correlations were observed between prototypical taste function (DTs and STs) and DTs and STs of both maltodextrin and oligofructose. Participants were stratified into tertile groups according to the complex carbohydrates and sweeteners tested and all taste measures. We observed that those who were able to detect maltodextrin in water at low concentrations (lower tertile; *n* = 8) were also more sensitive to oligofructose. Similarly, those who were able to detect maltodextrin in water at higher concentrations (higher tertile; *n* = 11) were also less sensitive to oligofructose. Interestingly, we also observed that five participants were more sensitive only towards complex carbohydrates (maltodextrin, oligofructose) but were less sensitive to caloric sweeteners (glucose, fructose, sucrose, erythritol).

**Table 5:** Detection thresholds for four prototypical tastants (% *w/v*), including mean, standard error of mean (SEM), and range.

	Detection Threshold	
	Mean ± SEM	Range
**Sodium chloride**	0.02 ± 0.002	0.02–0.07
**Citric acid**	0.015 ± 0.0004	0.013–0.025
**Caffeine**	0.007 ± 0.0002	0.006–0.009
**Monosodium glutamate (MSG)**	0.012 ± 0.0007	0.008–0.02

**Table 6:** Suprathreshold intensity ratings for four prototypical tastants on gLMS, given by mean and standard error of mean (SEM).

	Concentration (% *w/v*)	Mean ± SEM
**Sodium chloride**	0.6	16.7 ± 2.6
	1.2	24.4 ± 3.6
	2.3	32.8 ± 4.1
**Citric acid**	0.02	21.5 ± 4.7
	0.06	27.3 ± 4.7
	0.13	34.4 ± 4.9
**Caffeine**	0.02	11.6 ± 3.0
	0.04	19.8 ± 3.4
	0.08	30.3 ± 4.1
**Monosodium glutamate (MSG)**	0.05	11.6 ± 1.5
	0.10	18.0 ± 2.6
	0.20	22.5 ± 3.6

Seven participants were more sensitive towards maltodextrin but not to glucose. Similarly, six participants were more sensitive towards oligofructose but not to fructose. Looking at the concentrations, it is likely that they detected the complex carbohydrates in the sample rather than any free sugars in the complex carbohydrate sample. For example, one participant was able to detect maltodextrin at 0.04% *w/v* (Glucose: 0.0003% *w/v*, total sugars in maltodextrin: 0.0011% *w/v*) but only able to detect glucose at 1.1% *w/v*. Likewise, one participant was able to detect oligofructose at 0.04% *w/v* (Fructose: 0.0005% *w/v*, total sugars in oligofructose: 0.0012% *w/v*) but only able to detect fructose at 0.57% *w/v*. For STs, we observed that some participants experienced low intensity (lower tertile; *n* = 2) or high intensity (higher tertile; *n* = 5) for all complex carbohydrates and sweeteners measured. No participant was more sensitive and experienced high intensity or less sensitive and experienced low intensity to all complex carbohydrates and sweeteners tested across both measures (DTs and STs).

When participants were further stratified into tertile groups (DTs) according to the four taste primaries (sour, salty, bitter, umami) and complex carbohydrates, we observed that two participants (*n* = 2) were more sensitive towards all four-taste primaries. Similarly, some participants experienced high intensity (*n* = 4) or low intensity (*n* = 4) when stratified into tertile groups (STs) according to the four taste primaries (sour, salty, bitter, umami) and complex carbohydrates. No participant was more sensitive and experienced high intensity or less sensitive and experienced low intensity towards all four taste qualities and complex carbohydrates across both measures (DTs and STs).

## Discussion

Our data support the hypothesis that complex carbohydrates (maltodextrin, oligofructose) can be sensed in the oral cavity over a range of concentrations by human participants. Furthermore, our data predicate that oral sensitivity to complex carbohydrates (maltodextrin, oligofructose) is not related to DTs of sweeteners (and other prototypical tastants) but there is overlap with perceived sweetener intensities.

The prevailing understanding at present is that the human taste system is now widely accepted to include five basic tastes (sweet, sour, bitter, salty, and umami taste), and fat taste being accepted by a few [24, 26–35]. Nevertheless, fat taste does not appear to have the same perceptual salience as the other five basic taste qualities [24]. Rather, the reported “taste” resembling effects from orally perceivable fatty acids only appear to be true at a DT level (lowest level at which a difference can be detected) as fatty acids do not stimulate suprathreshold taste intensity perception like the other five primary taste qualities [24, 36]. Furthermore, intensity perception for long chain fatty acids is controversial as intensity may be a function of irritation, smell, or any textural sensation. In order for oral perception of complex carbohydrates to be classified as a taste component, certain criteria that have been proposed previously should be met [31, 37]. These criteria comprise the following: 1) provides an adaptive (evolutionary) advantage; 2) is elicited by a unique class of chemicals; 3) has an independent transduction mechanism; 4) signals are detected through gustatory nerves that are processed in the gustatory cortex; 5) is perceptible and has a unique sensation that does not overlap with any other prototypical taste qualities; and 6) raises a behavioural and/or physiological reaction [31, 37, 38]. In the following paragraphs, the discussion will consider the evidence supporting complex carbohydrate as a “taste” component related to each of these criteria.

In regards to complex carbohydrates, the evidence outlined in the present study provides support for two of the stipulated criteria for a taste primary, i.e., is elicited by a unique class of chemicals and perceptual independence (perceptual independence with sweet taste is at DT only, but overlap with sweetness at intensity). At present, our data provide evidence that complex carbohydrates (oligosaccharides: maltodextrin, oligofructose) are perceptible and there were no robust correlations observed between the four basic taste primaries (both DTs and STs) and DTs and STs of both complex carbohydrates (maltodextrin and oligofructose). For sweet taste, DTs of the complex carbohydrates (maltodextrin, oligofructose) and the sweeteners (glucose, fructose, sucralose, Rebaudioside A, and erythritol) were not correlated. However, there were moderate correlations between the STs of the complex carbohydrates and sweeteners. In light of our methodological approach, that is: a) participants were asked not to swallow any samples during testing, mouth rinsing with deionised water between tasting samples, and the use of nose clips to eliminate any orthonasal and retronasal olfaction cues; b) use of red lights to reduce any perceptual differences due to colour (visual) of samples; c) repeated testing of up to seven times per complex carbohydrate and good test-retest reliability of complex carbohydrates; d) a wide range of concentrations used starting from low concentration levels; and e) solutions were prepared fresh on the day, we are confident that the DTs and STs reported were unique to oral taste sensitivity to complex carbohydrates, and not based on additional orosensory cues such as olfaction and visual. However, the most challenging potential confound is with texture/viscosity and hydrolysis of complex carbohydrates by *α*-amylase which could result in liberation of free glucose in the tasting procedure, especially at higher concentration levels. While we observed that some participants were able to consistently differentiate complex carbohydrate solutions from water at the lowest concentration levels tested (0.04% *w/v*), still, the evidence is not conclusive that the DTs and STs reported were not due to additional textural cues. Interestingly, though, we used oligofructose which could not be hydrolysed by *α*-amylase and the present results between maltodextrin and oligofructose were highly correlated (both DTs and STs) indicating free glucose in the maltodextrin was not a significant confounding factor. Therefore, while not diminishing the prospect that oral complex carbohydrate sensitivity could be due to textural differences and oral perception of liberated free glucose, the present finding suggests that complex carbohydrates are perceptible in the oral cavity and have a distinct oral sensation that does not overlap with any primary taste qualities. These findings are consistent with Lapis et al. [4], where the STs of maltodextrin (DE5 and 10) were not significantly correlated to sodium chloride (salty taste), glucose (sweet taste) and sucrose (sweet taste). Furthermore, the present finding refutes the historical assumption that complex carbohydrates are tasteless to the human palate system [39–42].

One obstacle to acceptance of complex carbohydrate as a taste quality has been the identification of potential pathways and or receptor(s). At present, it is widely accepted that the sweet taste receptors are the only carbohydrate sensing receptors in the oral cavity. The primary sweet sensor, the sweet taste receptor consists of two heterodimer G-protein coupled receptors, the T1R2-TIR3 [43]. The T1R2 and T1R3 dimers entail a large extracellular area (i.e., Venus fly trap domain), which is connected to the transmembrane via a cysteine-rich domain [44]. It has been suggested that the cysteine-rich domains activate sweet proteins, whereas, the Venus flytrap domain of T1R2 targets a large variety of sweet substances (caloric sweeteners and most of the NNS) and the Venus flytrap domain of T1R3 targets other NNS, such as cyclamate and sweet receptor blocker, lactisole [44, 45]. A significant issue is whether or not complex carbohydrates are detected through the same taste receptor that detects sweetness (i.e., T1R2-T1R3 heterodimer). The present results showed that the discriminability of the caloric sweeteners (glucose, fructose, sucrose, and erythritol) from water were about the same, as were NNS (sucralose and Rebaudioside A). There were also strong correlations between the DTs of the complex carbohydrates (maltodextrin, oligofructose). However, the DTs of the complex carbohydrates and all of the sweeteners were not correlated, highlighting that mechanisms other than the T1R2-T1R3 are responsible for the detection of complex carbohydrates. Considering the concentrations used, it is possible that the participants detected the complex carbohydrates in the maltodextrin samples instead of the free sugars. The current data is consistent with the previous psychophysical studies where participants were found to be able to perceive complex carbohydrates (glucose polymer, glucose oligomers), and the sensitivity to simple sugar (glucose, sucrose) was independent of that to complex carbohydrates [4, 5]. In the study by Lapis et al. [5], it was found that humans (*n* = 25) were able to discriminate complex carbohydrate solutions (glucose oligomers) from water even when the sweet taste receptor (T1R2-T1R3 heterodimer) was inhibited by lactisole treatment–a sweet taste blocker known to bind to a pocket in the transmembrane region of the T1R3 and thus inhibits the sweet taste perception of sugars, proteins and NNS [9]. Remarkably, although Lapis et al. [4] observed large individual variances between participants in terms of *α*-amylase activity, taste responsiveness to maltodextrin (DE 20, 10, and 5) was not significantly different between groups of participants with high *α*-amylase activity and low *α*-amylase activity. The present study is also in line with the results of animal studies in which knockout mice missing functional genes for both components of the sweet taste receptor (heterodimer of T1R2 and T1R3) show no genetic, electrophysiological, and behavioural reactions to simple sugars (glucose, fructose, or sucrose) but respond normally to complex carbohydrates [46–52]. Besides, acceptability of complex carbohydrate (maltodextrin) was found to be unaccounted for by the small amount of free sugars (~0.05–2.88% *w/v* glucose and maltose) contained in maltodextrin, but rather, rodents appear to be highly attracted to the complex carbohydrate (maltooligosaccharide) itself [1, 53, 54]. Together, these findings raise the potential existence of an unidentified complex carbohydrate taste receptor in humans that responds to complex carbohydrates independently of those of sweet tastants [3].

Interestingly, at present, there were moderate correlations between the STs of complex carbohydrates and sweeteners. Potential explanation for this is that a novel receptor might still be involved in the transduction mechanism used to detect complex carbohydrates, but only for the detection range. At the perceptual range, the perception of complex carbohydrates (maltodextrin) could be partly mediated by the T1R-independent sweet sensing pathways in addition to the putative complex carbohydrate detection receptor (see discussion in Lapis et al. [4, 5]). It is also possible that the orally expressed enzymes such as salivary *α*-amylase, sucrose-isomaltase, and maltase-glucoamylase enzymes may locally break down dietary oligosaccharides, disaccharides, and starch hydrolysis products into monosaccharides [55]. Thus, the monosaccharides and free sugars in complex carbohydrates (maltodextrin) may combine to activate the T1R2-T1R3 sweet taste receptor and/or T1R-independent sweet pathway in taste receptor cells [55], which could explain the commonality seen with sweet taste in the perceptual range. However, at the detection range, the amount of free sugars in complex carbohydrates (maltodextrin) may be too low to activate conscious sweet taste perception [56]. Thus, this explanation may potentially explain why we only observed commonality with the sweet taste mechanism for the perceived intensity range, but not at the detection ranges. Given that orally expressed enzymes do not have any known effect in hydrolysing oligofructose, it is unknown at this stage why commonalities were observed between oligofructose and the sweeteners measured.

The finding that complex carbohydrates, maltodextrin and oligofructose, were strongly correlated with each other suggests some sort of similarity between both complex carbohydrates in terms of transduction pathways. We are uncertain why these similarities were observed given that oligofructose has been described to provide around thirty percent of the sweetness of sucrose and has been used in combination with NNS to replace sucrose in foods [57, 58]. Furthermore, the chemical structure is different between both of these complex carbohydrates and to our knowledge there does not appear to be any published animal data suggesting similarities in taste between maltodextrin and oligofructose. However, in studies investigating the effects of oligofructose on appetite profiles, maltodextrin was used as placebo supplements as they have been suggested to have a similar appearance and oral sensation as oligofructose [19, 20, 22]. It is also possible that similarities were observed between both complex carbohydrates in this study as they have a similar texture or mouthfeel, thus seeing commonalities between them.

There was large inter-individual variation in oral complex carbohydrate perception, and individuals may be classified as more or less sensitive to complex carbohydrates based on their sensitivity towards complex carbohydrates. For example, the concentration required to reach DT for maltodextrin varied 158 folds across the sample population. There was also large individual difference in perceived complex carbohydrate intensity. For example, the same maltodextrin sample (20% *w/v*) was rated 2.5 gLMS by one participant but 32.9 gLMS by another. Inter-individual differences or variability in taste function has also been previously observed for other taste qualities such as sweet [10, 59–61]. However, it is possible that large inter-individual differences in oral complex carbohydrate sensitivity were observed because of individual differences in AMY1 gene copy number and salivary *α*-amylase levels [62] but not taste. In this study, individuals with lower salivary amylase levels reported slower and significantly lesser decrease in perceived oral starch viscosity (oral viscosity thinning) in comparison to individuals with higher salivary amylase activity [62].

The current evidence from animal studies and human exercise studies provides support for the remaining stipulated criteria for oral complex carbohydrate sensitivity as a taste component (i.e., criteria 1, 4, and 6). Considering the evolutionary advantages of our taste system, it could be argued that the physiological regulation and functional significance of sensing low amounts of complex carbohydrate is beneficial to the survival of human beings, especially during times when foods are scarce as complex carbohydrates represent a major source of energy for body functioning [63]. The adaptive advantage of complex carbohydrate sensing in the oral cavity is supported with the behavioural evidence from animal studies where rodents prefer complex carbohydrate solutions to solutions containing simple sugars, especially at low equi-molar concentrations [39, 64]. In addition, Sclafani and Mann [65] found that the preference profiles for five different carbohydrates varies as a function of concentration in three minute two-bottle choice tests. For example, at low molar concentrations, rats preferred maltodextrin to sugars (maltose, sucrose, glucose, fructose), whereas at higher molar concentrations, rats preferred sucrose and maltose in comparison to maltodextrin [65]. In a recent study by Poole et al. [66] investigating the phenotypic differences among eight inbred strains of mouse, strain variation in complex carbohydrate (maltodextrin) perception that is distinct from variation in sweet (sucrose) perception has been observed. More recent physiological evidence from exercise science found that exercise performance significantly improved after participants rinsed their mouth with solutions containing complex carbohydrate (maltodextrin) compared to NNS control solutions. Similarly, these findings were also replicated by other exercise scientists [67–75]. Additionally, Chambers et al. [76] further investigated the cortical response to oral maltodextrin and glucose solutions, revealing a similar pattern of brain activation in response to both solutions, including brain areas believed to be involved in the reward system (i.e., activates brain reward centres in orbitofrontal cortex and striatum similar to oral glucose, which were unresponsive to NNS). Together, these findings provide strong behavioural and physiological evidence that there may be taste transduction pathways that respond to complex carbohydrate independently of those for sweet taste [69]. Supporting one of the six criteria for oral perception of complex carbohydrates to be classified as a taste component, one study by Vigorito et al. [77] provided evidence that there is some specialisation of function within the rat’s peripheral gustatory system in response to complex carbohydrates. The results of this study revealed that selective gustatory nerve transection of the chorda tympani nerve, glossopharyngeal nerve, greater superficial petrosal nerve, and the pharyngeal branch of the vagus nerve differentially altered the intake of sucrose and maltodextrin solutions [77]. Interestingly, gustatory denervation of all four gustatory nerves (chorda tympani, glossopharyngeal nerve, greater superficial petrosal nerve, and chorda tympani nerve) in rats reduced their intake of both sucrose and maltodextrin solutions by the same degree [77]. These results indicate that while the intake of sucrose and maltodextrin appeared to be facilitated to the same level by the gustatory system, the pathways involved appear to vary [1, 77].

The evidence outlined in the present paper provides support for each of the proposed criteria for a taste component. However, due to the limited studies conducted in humans, the evidence supporting most of the criteria is not conclusive and thus warrants further investigation. There are some limitations that need to be taken into account when considering the results. It is important to acknowledge that this study does not control for salivary *α*-amylase during sample testing. Salivary amylase has been shown to hydrolyse *α*-1, 4 glycosidic bonds once mixed with complex carbohydrates, resulting in changes in texture [62]. Thus, we were unable to rule out the possibility that participants experienced differences in oral complex carbohydrate sensitivity due to differences in texture instead of the “taste” component. Therefore, more evidence from tribology studies is required to ensure that the DTs and STs reported were not due to textural cues. Furthermore, we also cannot rule out hydrolysis by salivary *α*-amylase could have liberated free glucose in maltodextrin samples, especially at higher concentration levels.

## Conclusion

Contrary to the previous understandings of the human taste system where complex carbohydrates have long been assumed to be tasteless to the human palate, our data highlight that complex carbohydrates (maltodextrin, oligofructose) are perceptible in the oral cavity and have a distinct oral sensation that does not overlap with any primary taste qualities. Additionally, our data indicate that oral sensitivity to complex carbohydrate is not related to a range of sweeteners at low concentration levels (DTs). The findings are consistent with the proposition of an independent mechanism for complex carbohydrates, but only for lower concentration levels. At the perceptual range, it is possible that the perception of complex carbohydrates may be partly mediated by the T1R-independent sweet sensing pathways in addition to the putative complex carbohydrate detection receptor. Another possibility is that the taste cell expressed enzymes such as the salivary *α*-amylase enzymes may locally break down dietary oligosaccharides, disaccharides, and starch hydrolysis products into monosaccharides. Thus, the monosaccharides and free sugars in complex carbohydrates (maltodextrin) combine to activate the T1R2-T1R3 sweet taste receptor and/or T1R-independent sweet pathway in taste receptor cells thereby showing the commonality with sweet taste in the perceptual range. However, it is unknown at this stage why commonalities were observed between oligofructose and the sweeteners measured.
